# The Metabolic Signature of Cardiorespiratory Fitness: A Systematic Review

**DOI:** 10.1007/s40279-021-01590-y

**Published:** 2021-11-10

**Authors:** Justin Carrard, Chiara Guerini, Christian Appenzeller-Herzog, Denis Infanger, Karsten Königstein, Lukas Streese, Timo Hinrichs, Henner Hanssen, Hector Gallart-Ayala, Julijana Ivanisevic, Arno Schmidt-Trucksäss

**Affiliations:** 1 Division of Sports and Exercise Medicine, Department of Sport, Exercise and Health, University of Basel, Grosse Allee 6, CH-4052 Basel, Switzerland; 2 University Medical Library, University of Basel, Basel, Switzerland; 3 Metabolomics Platform, Faculty of Biology and Medicine, University of Lausanne, Quartier UNIL-CHUV, Rue du Bugnon 19, CH-1005 Lausanne, Switzerland

## Abstract

**Background:**

Cardiorespiratory fitness (CRF) is a potent health marker, the improvement of which is associated with a reduced incidence of non-communicable diseases and all-cause mortality. Identifying metabolic signatures associated with CRF could reveal how CRF fosters human health and lead to the development of novel health-monitoring strategies.

**Objective:**

This article systematically reviewed reported associations between CRF and metabolites measured in human tissues and body fluids.

**Methods:**

PubMed, EMBASE, and Web of Science were searched from database inception to 3 June, 2021. Metabolomics studies reporting metabolites associated with CRF, measured by means of cardiopulmonary exercise test, were deemed eligible. Backward and forward citation tracking on eligible records were used to complement the results of database searching. Risk of bias at the study level was assessed using QUADOMICS.

**Results:**

Twenty-two studies were included and 667 metabolites, measured in plasma (*n* = 619), serum (*n* = 18), skeletal muscle (*n* = 16), urine (*n* = 11), or sweat (*n* = 3), were identified. Lipids were the metabolites most commonly positively (*n* = 174) and negatively (*n* = 274) associated with CRF. Specific circulating glycerophospholipids (*n* = 85) and cholesterol esters (*n* = 17) were positively associated with CRF, while circulating glycerolipids (*n* = 152), glycerophospholipids (*n* = 42), acylcarnitines (*n* = 14), and ceramides (*n* = 12) were negatively associated with CRF. Interestingly, muscle acylcarnitines were positively correlated with CRF (*n* = 15).

**Conclusions:**

Cardiorespiratory fitness was associated with circulating and muscle lipidome composition. Causality of the revealed associations at the molecular species level remains to be investigated further. Finally, included studies were heterogeneous in terms of participants’ characteristics and analytical and statistical approaches.

**PROSPERO Registration Number:**

CRD42020214375.

**Supplementary Information:**

The online version contains supplementary material available at 10.1007/s40279-021-01590-y.

## Key Points

**Table:** 

A panel of unique lipid species were found to be associated with cardiorespiratory fitness.
The majority of circulating glycerolipids, acylcarnitines, and ceramides were negatively associated with cardiorespiratory fitness, highlighting their link to poor cardiometabolic health.
Specific glycerophosphocholines and cholesterol esters were found to be positively associated with cardiorespiratory fitness, featuring their roles in health maintenance.

## Introduction

Cardiorespiratory fitness (CRF), defined as the peak oxygen uptake, is a powerful health marker [1]. Importantly, the American Heart Association now recommends assessing it as a vital sign in clinical practice [1]. Indeed, CRF is inversely associated with an incidence of cancer, cardiometabolic diseases, and all-cause mortality [1–6]. Furthermore, improvement in CRF is associated with a reduced incidence of stroke, type 2 diabetes mellitus, dementia, and all-cause mortality [7–12].

Physiologically, CRF reflects the entire oxygen transport chain from its uptake in the lungs to its delivery to the mitochondria for energy production [1]. While the heritability of basal CRF and gains in CRF is around 50%, the mechanisms linking CRF with reduced morbidity and mortality remain largely unknown [1, 13, 14]. As CRF is a better predictor of morbidity and mortality than physical activity itself, physiological adaptations to exercise are likely not sufficient to explain how CRF mitigates morbidity and mortality [15–17]. Identifying metabolic signatures associated with CRF could reveal the metabolic pathways through which CRF acts on morbidity and mortality, lead to the discovery of novel biomarkers of physical fitness, and ultimately pave the way for novel health-monitoring strategies [18, 19].

In the past decade, technological advances in mass spectrometry, nuclear magnetic resonance, and bioinformatics have enabled ‘omics’ scale metabolite phenotyping [20]. Metabolomics is now a powerful tool to investigate at the molecular species level how metabolites relate to the cellular phenotype [21]. While genes encode *what may happen*, metabolites, influenced by both genome and exposome, provide insights on *what has indeed happened* [20]. Thus, the metabolome directly reflects cellular activity and is the closest ‘omic’ level to the phenome [20, 21]. Furthermore, metabolites not only constitute building blocks of cell components or fuels in cellular energetics, they also act as driving forces of cellular processes (e.g., cell growth, differentiation, activation, apopotosis) by modulating (through covalent chemical modifications or metabolite-macromolecule interactions) the expression and activity of the other ‘omics’ levels [22, 23]. Conversely, pathological processes can also alter both the metabolome and CRF as well as their mutual associations [24, 25]. Consequently, there are complex interrelated interactions between the genome, the metabolome, the exposome, and disease development, which all influence the phenome (e.g., CRF or health status) [20]. Considering the high clinical relevance of CRF, this work aimed at systematically reviewing the current literature on metabolites in human tissues and body fluids that have been reported to be associated with CRF.

## Methods

This systematic review is reported according to the Preferred Reporting Items for Systematic and Meta-Analysis (PRISMA) guidelines [26]. The research question was formulated according to the Population, Exposure, Comparison, Outcome, Study Type framework (Electronic Supplementary Material [ESM]) [27]. The review was registered on PROSPERO (registration number CRD42020214375) on 14 November, 2020 and a protocol was published [28].

### Eligibility Criteria

All human studies that (1) were published until the date of the last search, i.e., 3 June, 2021, (2) applied a metabolomics approach, (3) reported metabolites of any tissue, associated with CRF, and (4) measured CRF by means of a cardiopulmonary exercise test (spiroergometry) were eligible. Studies reporting estimated CRF were excluded as estimated CRF correlates only moderately with measured CRF [29]. Studies that were published in languages other than English, German, French, Italian, or Spanish were not included (as the authors have linguistic expertise in these five languages). Finally, non-original articles (i.e., editorials, letters, reviews), meta-analyses, case reports, and conference abstracts were also deemed non-eligible.

### Information Sources and Search Strategy

Search strategies were developed in collaboration with an information specialist (CAH) using the Peer Review of Electronic Search Strategies (PRESS) framework [30]. PubMed, Web of Science, and EMBASE were searched. Database-specific subject headings and text word synonyms around the concepts metabolomics and CRF were used. Non-human studies and conference abstracts were excluded. Search results were generated on 20 October, 2020, exported to EndNote X9 (Clarivate, London, UK) and deduplicated. An update search was run on 3 June, 2021. The detailed search strings can be found in the ESM and in the review protocol [28].

### Data Management and Extraction

Titles and abstracts of recovered records were reviewed independently by two authors (CG and JC). Articles were deemed as ‘include’, ‘exclude’ or ‘uncertain’ according to the prespecified eligibility criteria. For articles deemed ‘include’ or ‘uncertain,’ full text was retrieved and independently reviewed for eligibility by two authors (CG and JC). Discrepancies during title/abstract or full-text screening were resolved by discussion between the two screening authors. A third party made a final judgment in cases where no resolution was found (LS). To complement the results of direct database searching, bibliographic references of all included articles were screened manually (backward citation tracking), and the citing articles were screened using Scopus (forward citation tracking, on 15 June, 2021). Data were extracted from the full texts and entered in a standardized Excel form. One author extracted the data (CG), and a second author independently checked the extractions (JC). Discrepancies were resolved through discussion (with a third party, if necessary, LS). Corresponding authors were contacted twice by e-mail in cases of missing or unclear data. Information that was extracted can be found in the ESM.

### Risk of Bias in Individual Studies

The following key steps of a metabolomics workflow were extracted at the study level: sample collection and storage, sampling time and nutritional protocol, metabolite extraction method, analytical technique, quality control used to assess data quality, data processing, and metabolite annotation. Subsequently, two authors (CG and JC) independently assessed the risk of bias at the study level using the QUADOMICS items applicable to the present work (ESM) [31, 32]. Discrepancies were resolved through discussion (with a third party, if necessary, LS).

### Data Synthesis

Qualitative and quantitative data describing associations between metabolites and CRF were synthesized narratively and presented in a tabular and charted format. Metabolites were classified using the chemical taxonomy of the Human Metabolome Database (version 4.0) [33].

## Results

### Study Selection

**Fig. 1:**
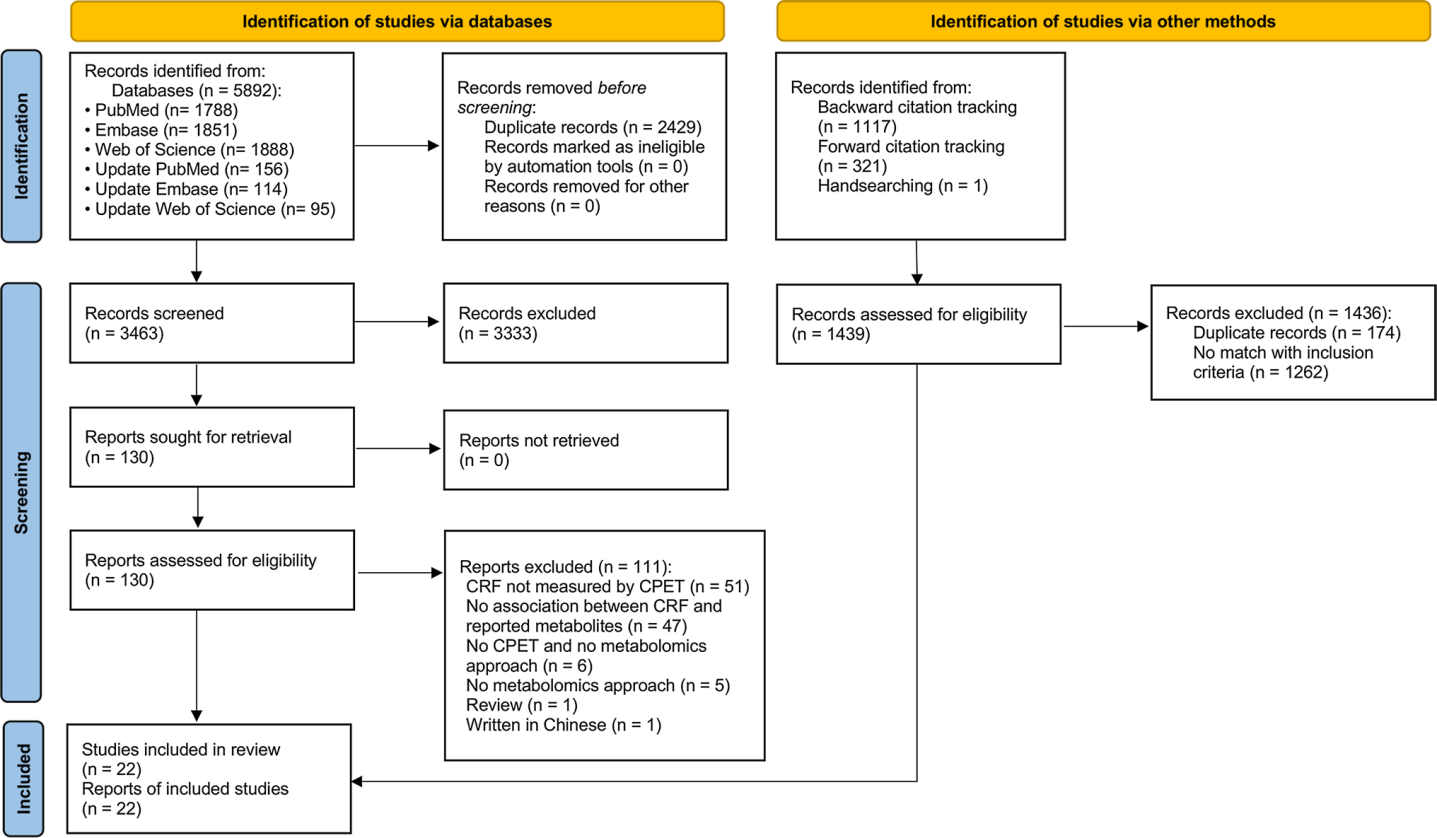
Preferred Reporting Items for Systematic and Meta-Analysis (PRISMA) 2020 flow diagram. *CPET* cardiopulmonary exercise test, *CRF* cardiorespiratory fitness

The searches yielded 4728 unique records, of which 22 met eligibility criteria as depicted in the PRISMA 2020 flow diagram (Fig. 1) [26]. Authors agreed on all eligibility decisions upon discussion without the need for third-party arbitration. Three additional studies were identified via other methods. One study was identified by a senior author (AST) via handsearching [34], one study was identified through backward citation tracking [35], and another study was identified through forward citation tracking, respectively [36].

### Characteristics of Included Studies

Included studies were published between 2012 and 2021 (Table 1 and ESM). Fifteen studies included both female and male participants [34–48], and seven studies investigated male participants only [49–55]. One study examined adolescents [38], and the remaining 21 studies enrolled only adults (defined as participants aged > 18 years).

**Table 1:** Studies’ and participants’ characteristics

References	Study design	Participants (*n*)	Age (Years ± SD)	Female sex (%)	VO_2_peak (mL/min/kg)	Health conditions	Tissue samples	Metabolomics approach	Technology used
Stanford et al. [37]	C	39	C1: 58.7 ± 2.5 C2: 29.4 ± 0.6	C1: 0 C2: 50	N/A	C1: overweight, obesity, arterial hypertension grade 1 C2: unknown smoking status	Serum	T (AQ)	LC–MS/MS
Duft et al. [38]	RCT	37	CG: 14.72 ± 1.07 TG: 14.44 ± 1.04	CG: 52 TG: 50	CG: 35.18 ± 6.22 TG: 32.80 ± 6.15	Overweight, obesity, unknown smoking status	Serum	T (AQ)	^1^H NMR
Saleem et al. [36]	L	100	64 ± 6	15	21.7 ± 5.5	Overweight, obesity, dyslipidemia, arterial hypertension, CAD, smoking, depression, musculoskeletal issues	Plasma	T (AQ)	HPLC–MS/MS
Kujala et al. [49]	CS	580	26.1 ± 6.5	0	HF: 50.7 ± 4.2 LF: 31.8 ± 3.8	Overweight, dyslipidemia, arterial hypertension, diabetes, smoking	Serum	T (AQ)	^1^H NMR
Fabbri et al. [34]	L	443	68.9 ± 9.4	42	24.2 ± 6.8	Dyslipidemia, arterial hypertension, prediabetes, diabetes, smoking	Plasma	T (AQ)	HPLC–MS/MS
Monnerat et al. [50]	P	14	HF: 25.8 ± 50.3 LF: 26.0 ± 5.0	0	HF: 76.3 ± 1.5 LF: 61.0 ± 3.5	None	Plasma	U	UHPLC-HRMS
Lustgarten et al. [39]	NRT	77	24.4 ± 4.2	64	44.4 ± 10.8	Overweight, obesity	Serum	U	UHPLC-MS/MS GC–MS
Harshman et al. [55]	POC	13	C1: 26.67 ± 5.16 C2: 29.43 ± 3.36	0	C1: 56.57 ± 10.40 C2: 43.03 ± 4.07	None	Sweat	T (RQ)	LC–MS/MS
Nayor et al. [41]	C	471	53.0 ± 8.0	63	23.1 ± 7.1	Overweight, dyslipidemia, arterial hypertension, diabetes, smoking	Plasma	T (AQ), U	LC–MS/MS
Contaifer et al. [40]	CS	49	57	25	14.0 ± 3.4	Overweight, obesity, dyslipidemia, arterial hypertension, diabetes, heart failure	Plasma	U	LC–MS/MS GC–MS
Mueller-Hennessen et al. [51]	CC	41	P: 51.5 ± 13.7 CG: 47.5 ± 12.8	0	P: 21.0 ± 8.0 CG: 32.0 ± 6.0	Overweight, dyslipidemia, arterial hypertension, diabetes, heart failure, smoking	Plasma	T (RQ)	LC–MS/MS GC–MS SPE-LC–MS/MS
Huffman et al. [42]	RCT	112	18–70	48	28.0 ± 5.8	Overweight, obesity, dyslipidemia, arterial hypertension grade 1	Skeletal muscle	T (AQ)	MS/MS GC–MS
Duft et al. [52]	RCT	22	CG: 47.50 ± 6.20 TG: 48.60 ± 5.50	0	CG: 29.10 ± 4.70 TG: 28.20 ± 4.70	Obesity grade 1	Serum	T (AQ)	^1^H NMR
Morris et al. [44]	RCT	40	35.0 ± 14.0	50	41.1 ± 16.2	Overweight, obesity	Plasma	T (AQ)	ESI–MS/MS
Contrepois et al. [45]	C	36	59.00 ± 8.00	42	30.60 ± 8.71	Overweight, arterial hypertension, prediabetes, diabetes, smoking	Plasma	U, ST	LC–MS/MS Lipidyzer Platform
Kistner et al. [46]	CS	255	46.1 ± 16.9	42	38.8 ± 11.6	None	Urine	T (AQ)	^1^H NMR
Chorell et al. [53]	I	27	HF TG: 28.16 ± 2.70 HF CG: 25.58 ± 1.77 LF TG: 26.30 ± 5.30 LF CG: 24.04 ± 1.83	0	HF TG: 63.20 ± 2.93 HF CG: 63.67 ± 2.80 LF TG: 44.57 ± 5.62 LF CG: 42.71 ± 2.87	None	Plasma	U	GC–MS/MS
Brennan et al. [47]	RCT	216	CG: 52.3 ± 8.4 TG: 52.4 ± 7.8	CG: 66 TG: 64	CG: 29.2 ± 6.0 TG: 28.4 ± 5.1	Overweight, obesity	Plasma	T (AQ)	LC–MS/MS
Morris et al. [43]	CC	65	HF: 28.0 ± 9.0 LF: 36.0 ± 11.0	48	HF: 54.9 ± 7.5 LF: 30.8 ± 7.2	Overweight, obesity	Urine	U	GC–MS
Shi et al. [54]	NRT	20	29.42 ± 4.51	0	59.20 ± 5.90	None	Serum	U	UHPLC-MS/MS
Bye et al. [48]	NRT	218	HF: 49.50 LF: 49.50	58	HF: 41.43 LF: 31.33	None	Serum	U	^1^H NMR
Michel et al. [35]	CC	40	P: 23.1 ± 5.1 CG: 24.7 ± 6.6	35	P: 28.8 ± 10.1 CG: 45.7 ± 6.4	Fontan patients with systemic left ventricle	Serum	T (AQ)	LC–MS/MS

*AQ* absolute quantification, *C1* cohort/group 1, *C2* cohort/group 2, *C* cohort, *CAD* coronary artery disease, *CC* case–control, *CG* control groups, *CS* cross-sectional, *ESI–MS/MS* electrospray ionization tandem mass spectrometry, *GC–MS* gas-chromatography tandem mass spectrometry, ^*1*^*H NMR* proton nuclear magnetic resonance, *HF* high fit, *HPLC–MS/MS* high-performance liquid-chromatography tandem mass spectrometry, *I* interventional, *L* longitudinal, *LC–MS/MS* liquid-chromatography tandem mass spectrometry, *LW* low fit, *MS/MS* tandem mass spectrometry, *N/A* not applicable, indicates that data were not reported in manuscripts and authors did not respond to our e-mail requests, *NRT* non-randomized trial, *P in the column “age" and “VO*_*2*_* peak”* patients, *P in the column study design* pilot, *POC* proof of concept, *RCT* randomized controlled trial, *RQ* relative quantification, *SD* standard deviation, *SPE-LC–MS/MS* solid-phase extraction liquid-chromatography tandem mass spectrometry, *ST* semi-targeted, *T* targeted, *TG* training/test group, *U* untargeted, *UHPLC-HRMS* ultra-high performance liquid-chromatography tandem high-resolution mass spectrometry, *VO*_*2*_*peak* peak oxygen uptake

Six studies included healthy participants free of any diseases [46, 48, 50, 53–55], of which one enrolled elite long-distance runners [50] and one enrolled amateur marathon runners [54]. In 14 studies, a subfraction or even all participants were overweight or obese [36–45, 47, 49, 51, 52]. Finally, in ten studies, a subfraction or even all participants had a cardiometabolic disease other than overweight or obesity [34–37, 40–42, 45, 49, 51].

Ten studies investigated plasma samples [34, 36, 40, 41, 44, 45, 47, 50, 51, 53], eight analyzed serum samples [35, 37–39, 48, 49, 52, 54], two investigated urine samples [43, 46], one analyzed skeletal muscle [42], and one investigated sweat [55]. Twelve studies applied a targeted approach [34–38, 42, 44, 46, 47, 49, 51, 52], and eight studies applied an untargeted approach [39, 40, 43, 48, 50, 53–55]. Depending on the nature of metabolites, one study used a targeted or untargeted approach [41], whereas another used a targeted or semi-targeted approach [45]. Fifteen studies ran regression analyses to investigate associations between metabolites and CRF [34, 36, 39–41, 43–45, 47–51, 53, 55], and seven studies conducted correlation analyses [35, 37, 38, 42, 46, 52, 54] (ESM).

### Risk of Bias in Individual Studies

Risk of bias assessment is summarized in Table 2. Sixteen studies failed to precisely describe the selection process of participants [34, 36, 37, 39, 40, 42–45, 48, 50–55], and 13 studies did not take any actions to avoid overfitting [34–36, 40–47, 50, 55]. While 18 studies collected tissue samples after an overnight fasting [34, 35, 37–39, 41–47, 49–54], Bye et al. [48] collected information on dietary habits without imposing fasting, Harshman et al. [55] collected sweat in a non-fasted state and two studies did not report on the nutritional state (ESM) [36, 40]. Harshman et al. [55] collected sweat during an exercise intervention, while the remaining 21 studies collected tissue samples in a resting state. Mueller-Hennessen et al. [51] did not specify the extraction protocol used. Finally, Lustgarten et al. [39] Huffman et al. [42] Kujala et al. [49] and Mueller-Hennessen et al. [51] did not report on the quality control used.

**Table 2:**
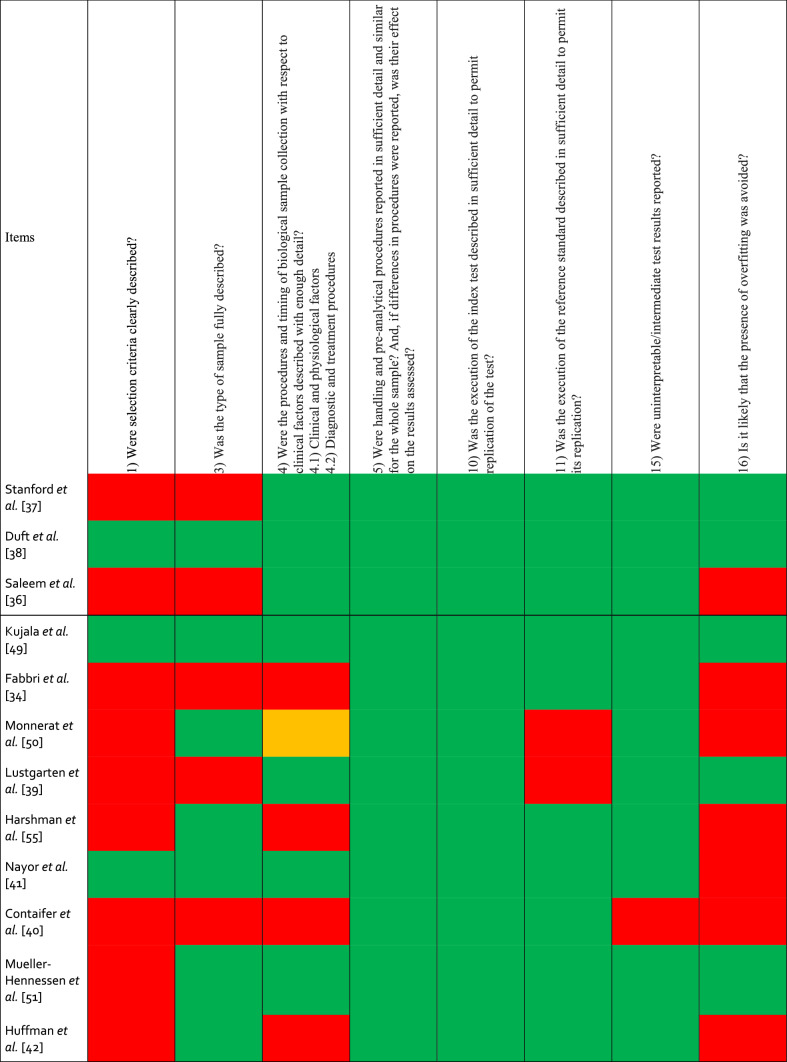

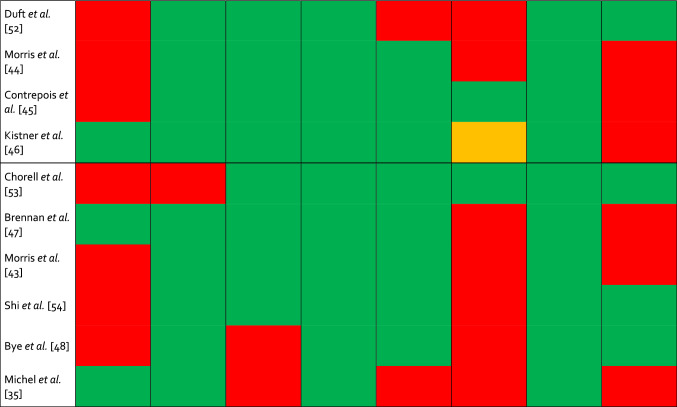
Risk of bias in individual studies evaluated with QUADOMICS

Green = yes, red = no, orange = unclear

### Metabolites Related to CRF

A total of 667 metabolites were reported to be significantly associated (*n* = 639) or correlated (*n* = 28) with CRF. These 667 metabolites were measured in plasma (*n* = 619), serum (*n* = 18), skeletal muscle (*n* = 16), urine (*n* = 11), or sweat (*n* = 3). Results are presented in a tissue-specific manner with plasma and serum metabolites being grouped together under the term circulating metabolites. Twenty-seven of the metabolites extracted from Nayor et al. [41] were doubly reported as per two different analytical techniques used for their detection (hydrophilic interaction liquid chromatography, positive ion mode analyses of polar and nonpolar plasma lipids using reversed-phase chromatography or negative ion mode analysis of free fatty acids and bile acids using reversed-phase chromatography). Similarly, Contrepois et al. [45] doubly reported four metabolites as they “eluted in multiple peaks”. As all these 31 metabolites were collected from plasma, they were counted only once in this analysis. Furthermore, two studies reported only on metabolites non-significantly associated [50] or correlated [35] with CRF. These metabolites were not considered in the present analysis.

#### Circulating Metabolites

**Fig. 2:**
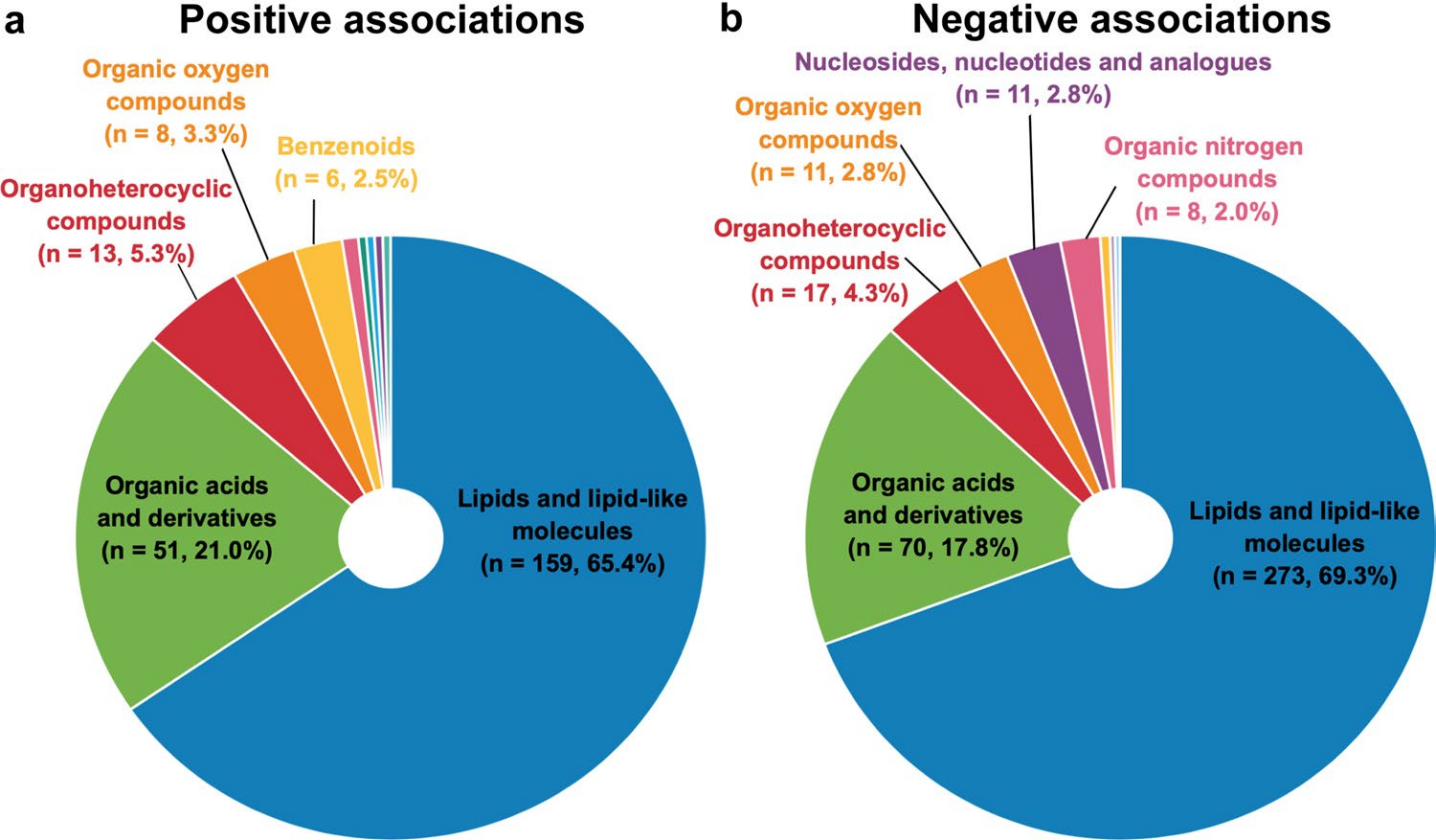
Included metabolites on the super class level. **a** Metabolites super classes positively associated with cardiorespiratory fitness. **b** Metabolites super classes negatively associated with cardiorespiratory fitness. *n* number of metabolite species. Figure was created with the Mind the Graph platform (www.mindthegraph.com) and Adobe Illustrator 2021 (Adobe Inc., San Jose, CA, USA)

Circulating metabolites were positively (*n* = 243) and negatively (*n* = 394) associated with CRF. As displayed in Fig. 2a, the former consisted of lipids and lipid-like molecules (*n* = 159), organic acids and derivatives (*n* = 51), organoheterocyclic compounds (*n* = 13), organic oxygen compounds (*n* = 8), benzenoids (*n* = 6), organic nitrogen compounds (*n* = 2), nucleosides, nucleotides, and analogs (*n* = 1), phenylpropanoids and polyketides (*n* = 1), lignans, neolignans, and related compounds (*n* = 1), and inorganic compounds (*n* = 1). As shown in Fig. 2b, metabolites negatively associated with CRF were subdivided into lipids and lipid-like molecules (*n* = 273), organic acids and derivatives (*n* = 70), organoheterocyclic compounds (*n* = 17), organic oxygen compounds (*n* = 11), nucleosides, nucleotides and analogs (*n* = 11), organic nitrogen compounds (*n* = 8), benzenoids (*n* = 2), phenylpropanoids and polyketides (*n* = 1), and alkaloids and derivatives (*n* = 1).

**Fig. 3:**
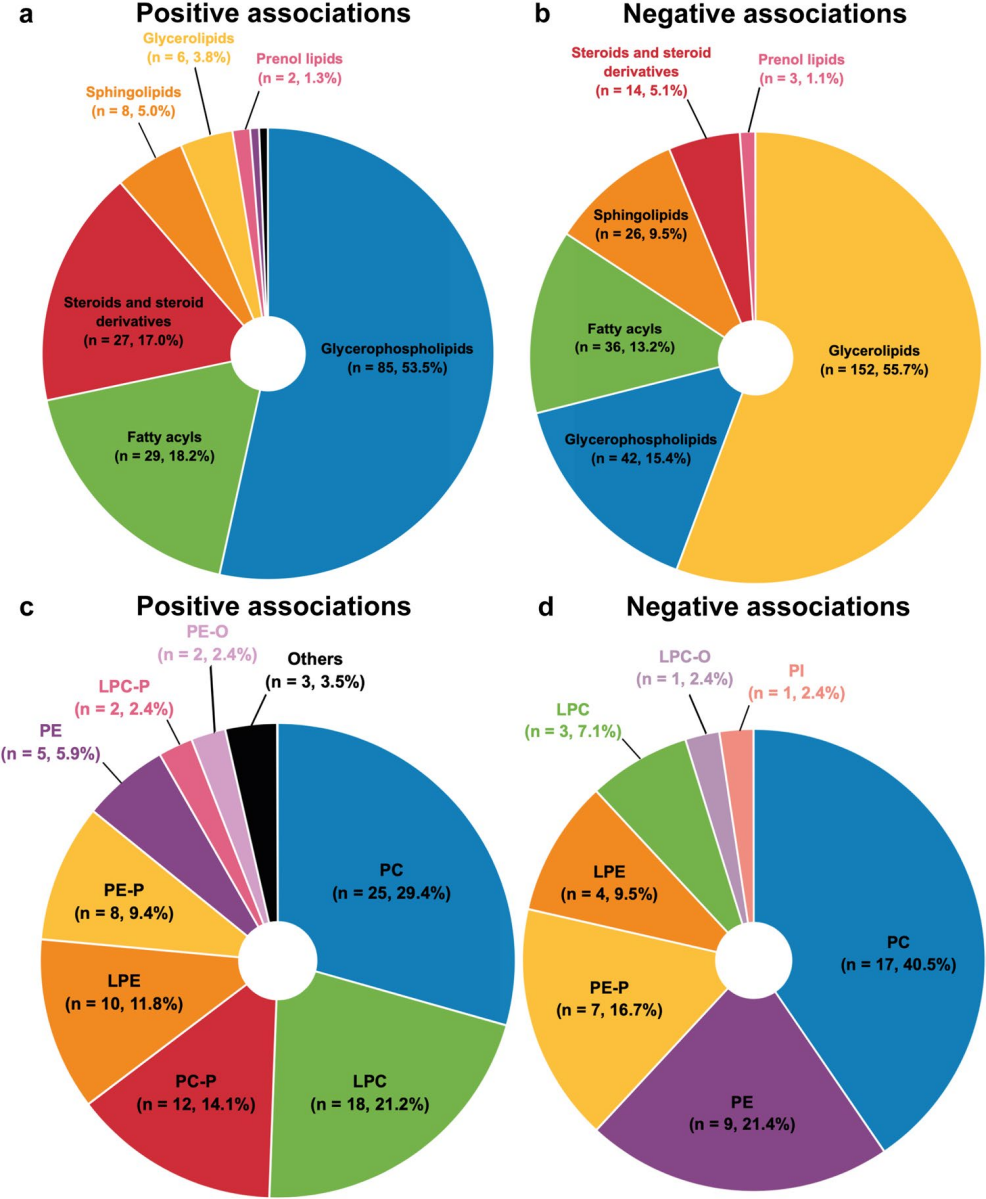
Included lipid and lipid-like molecules. **a** Lipids and lipid-like molecules positively associated with cardiorespiratory fitness, by class level. **b** Lipids and lipid-like molecules negatively associated with cardiorespiratory fitness, by class level. **c** Glycerophospholipids positively associated with cardiorespiratory fitness, by direct parent level. **d** Glycerophospholipids negatively associated with cardiorespiratory fitness, by direct parent level. *LPC* lyso-acylglycerophosphocholines, *LPC-O* lyso-alkylglycerophosphocholines, *LPC-P* lyso-alkenylglycerophosphocholines, *LPE* lyso-acylglycerophosphoethanolamines, *n* number of metabolite species, *PC* diacylglycerophosphocholines, *PC-P* alkenyl-acylglycerophosphocholines, *PE* diacylglycerophosphoethanolamines, *PE-O* alkyl-acylglycerophosphoethanolamines, *PE-P* alkenyl-acylglycerophosphoethanolamines, *PI* diacylglycerophosphoinositol. Figure was created with the Mind the Graph platform (www.mindthegraph.com) and Adobe Illustrator 2021 (Adobe Inc., San Jose, CA, USA)

Among the 159 lipids and lipid-like molecules positively associated with CRF, glycerophospholipids (*n* = 85), fatty acyls (*n* = 29), steroids and derivatives (*n* = 27), sphingolipids (*n* = 8), and glycerolipids (*n* = 6) were the most prevalent lipid classes (Fig. 3a). Glycerophospholipids were further subdivided into mainly diacylglycerophosphocholines (PC, *n* = 25), lyso-acylglycerophosphocholines (LPC, *n* = 18), alkenyl-acylglycerophosphocholines (*n* = 12), lyso-acylglycerophosphoethanolamines (*n* = 10), and alkenyl-acylglycerophosphoethanolamines (*n* = 8) (Fig. 3c). Fatty acyls comprised very long-chain fatty acids (*n* = 7), long-chain fatty acids (*n* = 7), medium-chain fatty acids (*n* = 5), and acylcarnitines (*n* = 4). Steroids and derivatives mainly consisted of cholesterol esters (*n* = 17). Lastly, the sphingolipids consisted of sphingomyelins (*n* = 5), glycosphingolipids (*n* = 2), and ceramides (*n* = 1).

As shown in Fig. 3b, the 273 lipid species negatively associated with CRF consisted of glycerolipids (*n* = 152), glycerophospholipids (*n* = 42), fatty acyls (*n* = 36), sphingolipids (*n* = 26), steroids and derivatives (*n* = 14), and prenol lipids (*n* = 3). Glycerolipids consisted of triacylglycerols (*n* = 122) and diacylglycerols (*n* = 30). Glycerophospholipids consisted mainly of PC (*n* = 17), PE (*n* = 9), alkenyl-acylglycerophosphoethanolamines (*n* = 7), and lyso-acylglycerophosphoethanolamines (*n* = 4) (Fig. 3d). Fatty acyls consisted of acylcarnitines (*n* = 14), further classified into short-chain (*n* = 8), medium-chain (*n* = 3), and long-chain (*n* = 3) acylcarnitines, and long-chain fatty acids (*n* = 10). Sphingolipids consisted mainly of ceramides (*n* = 12), sphingomyelins (*n* = 7), and glycosphingolipids (*n* = 6). Last, steroids comprised bile acids and derivatives (*n* = 7), sulfated steroids (*n* = 5), and cholesterol esters (*n* = 2).

**Fig. 4:**
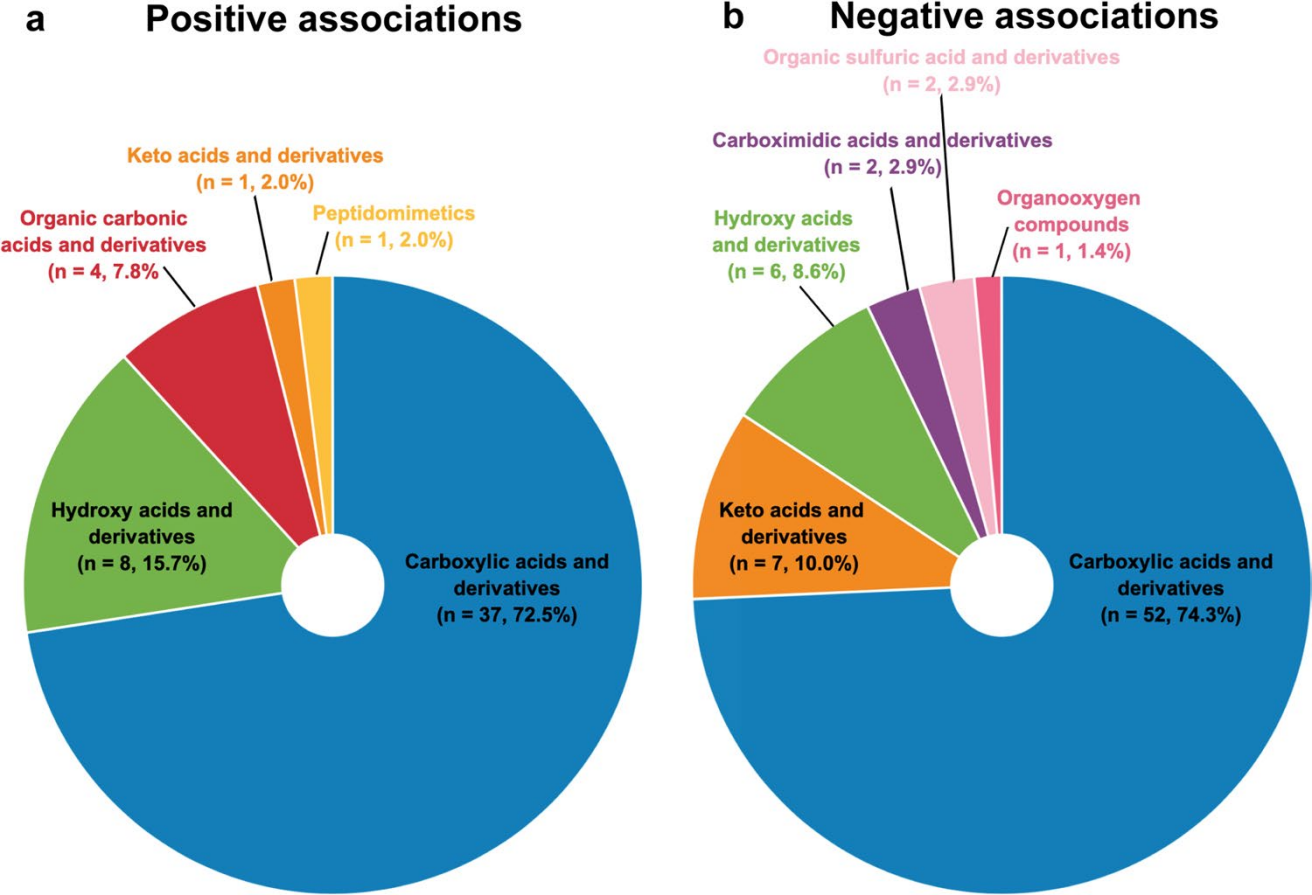
Included organic acids and derivatives on the class level. **a** Organic acids and derivatives positively associated with cardiorespiratory fitness. **b** Organic acids and derivatives negatively associated with cardiorespiratory fitness. *n* number of metabolite species. Figure was created with the Mind the Graph platform (www.mindthegraph.com) and Adobe Illustrator 2021 (Adobe Inc., San Jose, CA, USA)

The 51 organic acids and derivatives positively associated with CRF comprised 35 amino acids, peptides, and analogs, of which the most common were alpha amino acids and derivatives (*n* = 9), N-acyl-alpha amino acids (*n* = 4), methionine and derivatives (*n* = 3), prolines and derivatives (*n* = 3), and dipeptides (*n* = 3) (Fig. 4a). Seventy organic acids and derivatives were negatively associated or correlated with CRF, of which 51 were amino acids, peptides, and derivatives. The most represented amino acids and analogs were alpha amino acids and derivatives (*n* = 16), dipeptides (*n* = 5), arginines and derivatives (*n* = 4), tyrosine and derivatives (*n* = 4), prolines and derivatives (*n* = 3), and phenylalanines and derivatives (*n* = 3) (Fig. 4b).

The 13 organoheterocyclic compounds, which showed positive associations with CRF, consisted mainly of bilirubins (*n* = 4). Purines and purine derivates were the most represented metabolites (*n* = 4) within the 17 organoheterocyclic compounds negatively associated with CRF. Carbohydrates and carbohydrate conjugates were the most common metabolites within the eight organic oxygen compounds positively associated with CRF (*n* = 5) and in the 11 negatively associated with CRF (*n* = 8). Eleven nucleosides, nucleotides, and analogs displayed negative associations with CRF, while only one showed a positive association with CRF. Similarly, more organic nitrogen compounds were negatively associated with CRF (*n* = 8) than positively (*n* = 2). Finally, six benzenoids species displayed positive associations with CRF, while only two showed negative associations with CRF.

#### Metabolites Sampled from Skeletal Muscle, Urine, or Sweat

In skeletal muscle, 15 acylcarnitines were positively correlated to CRF, further classified into medium-chain (*n* = 9), long-chain (*n* = 5), and short-chain (*n* = 1) acylcarnitines. In urine, two metabolites (creatinine and uracil) were positively associated with CRF, while eight amino acids and analogs and one straight-chain fatty acid were negatively associated with CRF. Finally, sweat methionine showed a positive association with CRF, while sweat ornithine and phenylalanine displayed negative associations with CRF.

### Metabolites Reported Multiple Times in Relation to CRF

Ninety-seven circulating metabolites were reported by two or three distinct studies. Conflicting reports were found for 14 metabolites, which were reported to be either positively or negatively associated or correlated with CRF depending on the studies (Table 3). Fifteen metabolites, including four LPC species, were reported multiple times to be positively associated with CRF (Table 4). Finally, 68 metabolites were consistently negatively associated with CRF (Table 5). The latter included 44 triacylglycerols, nine amino acids, and three ceramides.

**Table 3:** Metabolite species reported to be positively and negatively associated with CRF

Metabolite super classes	Metabolite species	Studies reporting associations with CRF	
		Positive	Negative
Lipids and lipid-like molecules	Nervonic acid	[41]	[45]
	Vitamin A	[41]	[45]
	CE(20:4)	[41]	[45]
	CE(22:4)	[41]	[40]
Organic acids and derivatives	Pyroglutamic acid	[47]	[41]
	Histidine	[45]	[47]
	Methionine	[40]	[47]
	Cinnamoylglycine	[41]	[45]
	Phenylalanine	[52]	[41]
	Serine	[45]	[40]
	Tyrosine	[52]	[41, 47]
Organic oxygen compounds	1,5-Anhydroglucitol	[45]	[47]
	Kynurenine	[45]	[47]
Organoheterocyclic compounds	C-Glycosyltryptophan	[45]	[41]

*CE* cholesterol ester, *CRF* cardiorespiratory fitness

**Table 4:** Metabolites species reported multiple times as positively associated with CRF

Metabolite super classes	Metabolite species	Studies reporting positive associations with CRF
Benzenoids	Hippuric acid	[41, 45]
Lipids and lipid-like molecules	12,13-diHOME	[37, 41]
	Docosahexaenoic acid	[41, 53]
	PC(34:2)	[41, 44]
	LPC(18:0)	[41, 51]
	LPC(18:1)	[41, 51]
	LPC(18:2)	[41, 51]
	LPC(20:4)	[41, 51]
	SM(18:1;2/24:1)	[41, 45]
	CE(18:3)	[41, 45]
	CE(20:3)	[41, 45]
Organic acids and derivatives	Asparagine	[41, 45]
	Acetylglycine	[41, 45]
	Malic acid	[41, 47]
Organoheterocyclic compounds	Bilirubin	[41, 45, 47]

*CE* cholesterol ester, *CRF* cardiorespiratory fitness, *LPC* lyso-acylglycerophosphocholines, *PC* diacylglycerophosphocholines, *PC-P* alkenyl-acylglycerophosphocholines, *SM* sphingomyelin

**Table 5:** Metabolite species reported multiple times as negatively associated with CRF

Metabolite super classes	Metabolite species	Studies reporting negative associations with CRF
Lipids and lipid-like molecules	CAR(3) propionylcarnitine	[41, 45, 47]
	CAR(6) hexanoylcarnitine	[41, 45]
	TAG(46:1), TAG(46:2), TAG(46:3), TAG(47:0), TAG(47:2), TAG(48:1), TAG(48:2), TAG(48:3), TAG(48:4), TAG(48:5), TAG(49:0), TAG(49:1), TAG(49:2), TAG(49:3), TAG(50:1), TAG(50:2), TAG(50:3), TAG(50:4), TAG(50:5), TAG(50:6), TAG(51:0), TAG(51:1), TAG(51:2), TAG(51:3), TAG(52:1), TAG(52:2), TAG(52:3), TAG(52:4), TAG(52:5), TAG(52:6), TAG(52:7), TAG(53:2), TAG(53:3), TAG(54:1), TAG(54:2), TAG(54:3), TAG(54:4), TAG(54:5), TAG(54:6), TAG(54:7), TAG(54:8), TAG(55:2), TAG(55:3), TAG(56:5)	[41, 45]
	γ-Tocopherol	[39, 53]
	Cer(18:1;2/16:0)	[36, 41, 45]
	Cer(18:1;2/18:0)	[34, 36]
	Cer(18:1;2/20:0)	[34, 36]
	HexCer(18:1;2/18:0)	[36, 45]
	SM(18:1;2/18:0)	[41, 45]
	SM(18:1;2/18:1)	[36, 41, 45]
	Glycocholic acid	[41, 45]
Nucleosides, nucleotides, and analogs	Pseudouridine	[41, 51]
Organic acids and derivatives	Alanine	[41, 47]
	Citrulline	[45, 47]
	Creatine	[41, 45, 47]
	Lysine	[40, 41, 47]
	Ornithine	[41, 45, 47]
	Isoleucine	[41, 47]
	Leucine	[41, 47]
	Thyroxine	[41, 47]
	Proline	[41, 47]
	Lactic acid	[40, 41]
	Pyruvic acid	[41, 47]
Organic nitrogen compounds	Carnitine	[41, 45]
Organic oxygen compounds	Gluconic acid	[40, 45]

*CAR* carnitine, *Cer* ceramide, *CRF* cardiorespiratory fitness, *HexCer* hexosylceramide, *SM* sphingomyelin, *TAG* triacylglycerol

### Meta-Analysis

None of the included metabolites fulfilled the criteria, previously described in the review protocol, to be meta-analyzed [28]. Metabolites had (1) to be detected in the same tissue, (2) using the same metabolomics approach (untargeted, semi-targeted, or targeted), (3) in at least three different studies, and (4) to be identified on a level 1 identification according to the Metabolomics Standards Initiative to be meta-analyzed [28, 56].

## Discussion

**Fig. 5:**
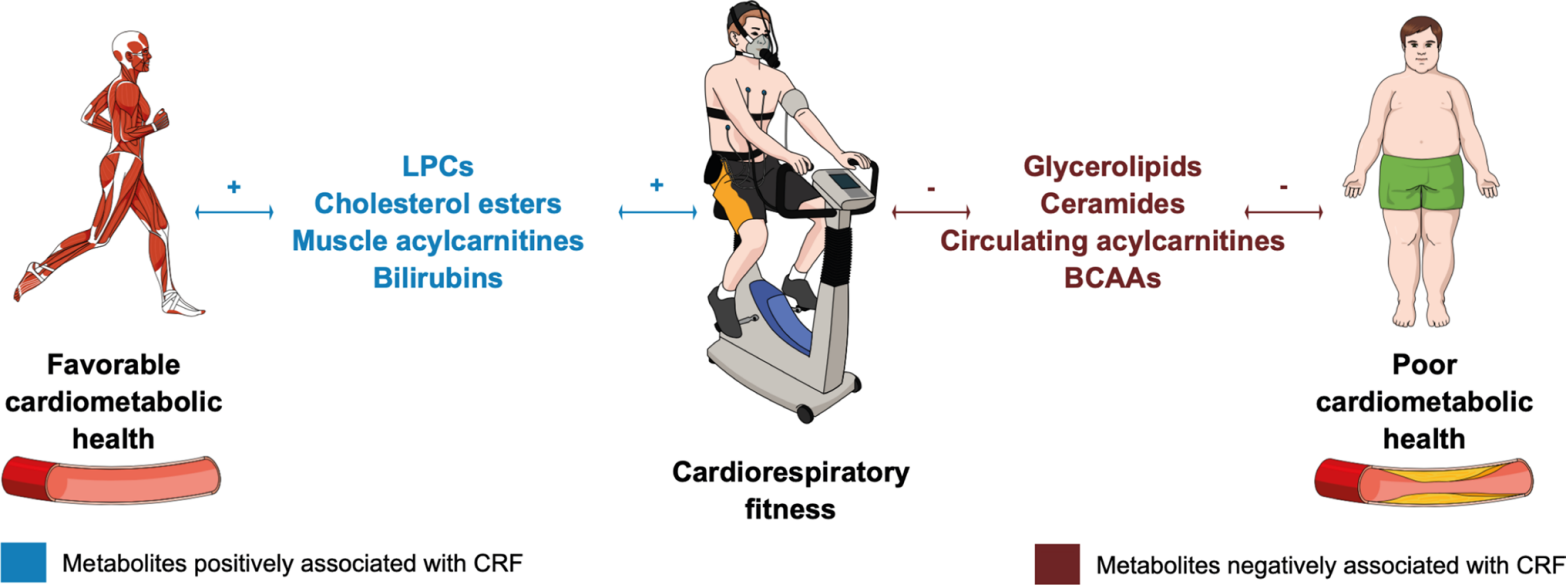
Metabolites associated with cardiorespiratory fitness (CRF) as potential driving forces of cardiometabolic health. Figure was created with the Mind the Graph platform (www.mindthegraph.com) and Adobe Illustrator 2021 (Adobe Inc., San Jose, CA, USA). *BCAA* branched-chain amino acids, *LPCs* lyso-acylglycerophosphocholines

The present work systematically reviewed metabolites that are associated with CRF, a potent marker of human health that should be considered as a vital sign in clinical medicine according to the American Heart Association [1]. Lipids, followed by organic acids, were the metabolites most commonly associated with CRF. Most circulating glycerolipids, acylcarnitines, and ceramides, as known biomarkers of poor cardiometabolic health, showed negative associations with CRF [57–63]. Conversely, most LPC and cholesterol esters were positively associated with CRF (Fig. 5). Branched-chain amino acids (BCAAs) were negatively associated with CRF, while bilirubin displayed positive associations with CRF (Fig. 5). None of the included metabolites fulfilled the qualitative and quantitative criteria defined in the review protocol to be meta-analyzed, which reflects the novelty of the present field. Nevertheless, 83 associations were reported independently by distinct studies, which strengthens the certainty of evidence of these findings. Conversely, conflicting results were found for 14 associations, which reduces their certainty of evidence. The following sections discuss the overall results with a focus on the 83 associations showing the strongest certainty of evidence.

### Lipids and Lipid-Like Molecules

In addition to their well-known functions in energy storage and production, lipids from biological membranes modulate cell surface receptor activities and regulate vesicular trafficking [64–66]. Lipids also act as key signaling molecules, controlling important cellular processes such as cell proliferation, apoptosis, migration, senescence, and inflammation [67, 68]. Alterations in lipid metabolism have been observed in many cardiometabolic, oncological, and neurodegenerative disorders [69, 70]. Remarkably, changes in the lipidome composition have been associated with aging, healthy aging, and age-related disorders [71–74]. Estimated to number in the hundreds of thousands of discrete molecular species, lipids are also the most abundant circulating macromolecules in human plasma [75]. In light of these facts, it is not surprising that lipids were the metabolites most commonly associated with a potent health marker such as CRF.

Specific PC species were previously described as cardiometabolically favorable, while others seem to be metabolically deleterious [62, 63, 76]. For instance, the favorable PC(16:0/22:5) and deleterious PC(16:0/16:0) are part of the ceramide-phospholipid score for the prediction of cardiovascular risk [62, 63], but the exact underlying biological mechanism remains unknown. Interestingly, saturated and monounsaturated PC species were previously positively associated with cardiovascular mortality [76]. The results of the present review support a dual role of PC species with 25 and 17 species being positively or negatively associated with CRF, respectively. No pattern was observed regarding species saturation and their association with CRF. Specifically, PC(34:2) was reported twice to be positively associated with CRF, which contrasts with the fact that this species was previously associated with cardiovascular mortality and aging [76, 77]. However, the annotation PC(34:2) does not unequivocally specify the fatty acyl or alkyl chains in the molecule, which makes data interpretation ambiguous [78]. Thus, PC(34:2) could correspond to different species, such as PC(16:0/18:2) but also PC(14:0/20:2), each of which have potentially different biological roles. Therefore, caution is necessary when interpreting lipidomic data.

Regarding LPC, more species were positively (*n* = 18) than negatively (*n* = 3) associated with CRF and four species [LPC(18:0), LPC(18:1), LPC(18:2), and LPC(20:4)] were reported twice as positively associated with CRF [41, 51]. LPC(18:0) and LPC(18:2) were previously associated with reduced cardiovascular mortality [76]. Mechanistically, LPCs are believed to inhibit cholesterol synthesis in macrophages and slow down atherogenesis [79]. LPC(16:0), which was reported once to be positively associated with CRF, is known to be inversely associated with vascular remodeling (intima-media thickness), cardiovascular diseases, and mortality [76, 80]. Finally, circulating LPC levels have been observed to be reduced in rodents with obesity and type 2 diabetes [81].

Twelve alkenyl-acylglycerophosphocholines species were found to be positively associated with CRF. Alkenyl-acylglycerophosphocholines belong to the ether-glycerophospholipid family, which acts, amongst other things, as cellular antioxidants and is therefore considered as metabolically favorable [82, 83]. This could explain why most alkenyl-acylglycerophosphocholines were positively associated with CRF. Additionally, lower circulating levels of ether-glycerophospholipids have been observed in patients with non-alcoholic steatohepatitis and children with type 1 diabetes [84, 85].

Circulating CE levels have been reported to be negatively associated with cardiovascular diseases [86, 87]. Indeed, the formation of cholesterol esters prevents intracellular free cholesterol accumulation [88]. The fact that 17 CE were positively associated with CRF, while only two CE were negatively associated with CRF supports this statement. On the lipid species level, CE(18:3) and CE(20:3) were reported twice as positively associated with CRF [41, 45]. Furthermore, CE(20:4), CE(20:5), CE(22:4), CE(22:5), and CE(22:6), levels of which were previously inversely associated with cardiovascular disease, displayed positive associations with CRF [86]. Most circulating acylcarnitines (regardless of chain length) were negatively associated with CRF, which reflects the fact that an accumulation of circulating acylcarnitines indicates incomplete mitochondrial fatty acid oxidation, and therefore impaired metabolic health [59, 60]. Conversely, muscular acylcarnitines were positively associated with CRF, highlighting improved fatty acid oxidation with a higher fitness level [89]. This is in line with previous data, which showed that exercise improves muscle mitochondrial capacity and the completeness of fatty acid oxidation [90].

Ceramides and their roles in cardiometabolic diseases are receiving growing scrutiny [91, 92]. On a mechanistic level, circulating ceramides are believed to promote foam cell formation, vascular inflammation, and atherosclerosis [93–95]. These findings have progressed to clinical medicine, where ceramides are now used to predict cardiovascular death in patients with and without coronary artery disease [61, 62, 96, 97]. Thus, it is postulated that ceramides act as driving forces of cardiometabolic disorders [98, 99]. In this context, the results of the present review are highly interesting, showing that 12 ceramides were negatively associated with CRF, with only Cer(18:1;2/10:0) being an exception. Furthermore, Cer(18:1;2/16:0) [36, 41, 45], Cer(18:1;2/18:0) [34, 36], and Cer(18:1;2/20:0) [34, 36] were reported several times as negatively associated with CRF. Strikingly, the three cardiometabolically deleterious ceramide species clinically used in the ceramide-phospholipid score (Cer(18:1;2/16:0) [36, 41, 45], Cer(18:1;2/18:0) [34, 36], and Cer(18:1;2/24:1) [36]) were found to be negatively associated with CRF [99]. In light of the these findings, it can be hypothesized that specific CRF-enhancing training could reverse altered ceramide profiles and optimize cardiometabolic health. This needs to be demonstrated in a prospective intervention study.

### Organic Acids and Derivatives

Amino acids not only serve as building blocks for proteins but also as signaling molecules, regulators of gene expression, as well as precursors of hormones and neurotransmitters [100]. Circulating levels of the BCAAs isoleucine, leucine, and valine have been associated with obesity, insulin resistance, and type 2 diabetes [101–104]. Initially attributed to an BCAA-mediated activation of the mammalian target of rapamycin pathway [105], these findings are more likely due to an increased ratio of BCAAs to tryptophan and threonine, resulting in central serotonin depletion, hyperphagia, obesity, and a reduced lifespan [106]. Alternatively, it has been suggested that metabolically healthy and cardiorespiratory fit individuals tend to have more efficient BCAA catabolism and fatty acid oxidation, which prevents BCAA accumulation in the circulation [107, 108]. The findings of the present systematic review tend to support the latter as isoleucine [41, 47], leucine [41, 47], and valine [47] were found to be negatively associated with CRF. While asparagine and acetylglycine are known to be inversely associated with the incidence of metabolic syndrome, these two amino acids were reported by distinct studies to be positively associated with CRF [109]. Phenylalanine and tyrosine are known to be elevated in subjects with insulin resistance, diabetes, or coronary artery disease [110]. Both metabolites also displayed higher levels in metabolically unhealthy obese patients, while they were not elevated in metabolically healthy obese subjects [111]. Remarkably, phenylalanine in plasma [41], urine [43], and sweat [55] as well as tyrosine in plasma [41, 47] and urine [46] were negatively associated with CRF. However, the contrary was true for phenylalanine and tyrosine in serum [52].

### Other Organic Compounds

Bilirubin, which was positively associated with CRF, is believed to be cardiometabolically favorable. Indeed, elevated bilirubin levels are associated with a reduced incidence of peripheral artery disease and stroke [112]. Conversely, low bilirubin levels have been associated with a higher risk of coronary artery disease, impaired flow-mediated vasodilatation, and increased carotid intima-media thickness [113, 114]. Therefore, elevation of bilirubin levels might be a way through which improvement in CRF mitigates the incidence of cardiometabolic diseases. Hippurate, the levels of which are decreased in patients with metabolic syndrome, was positively associated with CRF [115–117]. Finally, while circulating cell-free nucleic acids are increasingly recognized as potential biomarkers of diseases (‘liquid biopsy’) [118], little is known about the significance of isolated circulating nucleosides, which were mainly negatively associated with CRF [41, 45, 47].

### Strengths and Limitations

The present work was the first to systematically review CRF-associated metabolites. In this way, it provides researchers with an objective overview of the current literature and could orientate future research aiming at unraveling metabolic pathways through which CRF mitigates morbidity and mortality. The main limitation of the present systematic review lies in the important heterogeneity of the included studies. Indeed, studies differed in terms of participants’ age, sex, health conditions and medications, body mass index and percentage of body fat, physical activity, and CRF levels. Moreover, fasting protocols, analyzed tissues, sample preparation, extraction methods, analytical techniques, and metabolomics approaches were also heterogeneous. Furthermore, several statistical analyses were used, from simple correlations to multiple linear regressions, adjusting or not for important confounders. Therefore, investigating associations between metabolites and CRF needs to be done in healthy participants before investigating clinical populations. It will then be possible to circumvent the confounding effects of chronic cardiometabolic diseases on lipid metabolism. Second, some associations between metabolites and CRF are likely sex specific and, for female individuals, depend on menopausal status [119]. Unfortunately, data gathered from the included studies and from author contacts did not allow us to analyze associations in a sex-dependent or menopause-dependent manner. Indeed, seven studies investigated male individuals only [49–55], ten publications neither reported sex-specific or menopause-specific results nor provided individual patient data [36–38, 40–42, 44, 46–48], three studies reported sex-specific results without providing individual patient data [34, 39, 43], and two publications provided individual patient data without reporting sex-specific results [35, 45]. Coupled with the important heterogeneity described above, the lack of individual patient data prevented the creation of models predictive of CRF based on a metabolic signature.

Third, this systematic review does not provide information about the cellular origin, destination, or subcellular localization of the circulating pool of metabolites. Thus, potential CRF-promoting nutritional recommendations require further mechanistic studies in model organisms and intervention studies in both model organisms and humans. Consequently, such recommendations cannot be derived at this stage. Fourth, it is essential to harmonize analytical approaches by following recommendations edited by the Metabolomics Society [120, 121]. For instance, analyte concentration in sweat can vary greatly depending on the collection, handling, processing, storage, and skin microbiome [122, 123]. Finally, it is important to conduct regression analyses rather than simple correlation analyses and thereby adjust for relevant confounders [90].

## Conclusions

Circulating and muscle lipidome composition was associated with CRF, a clinically highly relevant health parameter. Known biomarkers of poor cardiometabolic health such as circulating glycerolipids, acylcarnitines, and ceramides were negatively associated with CRF. Conversely, circulating LPCs, cholesterol esters, and muscle acylcarnitines were positively associated with CRF, featuring their roles in health maintenance. BCAA and bilirubins showed negative and positive associations with CRF, respectively. It is important to note that the included studies were heterogeneous in terms of participants’ characteristics and analytical and statistical approaches. While causality of the revealed associations remains to be investigated further, lipid metabolism and changes in lipidome composition seem to be tightly related to physical fitness. Deciphering lipid responses to CRF-enhancing interventions could help unravel the metabolic pathways through which CRF mitigates morbidity and mortality.

## Supplementary Information

Below is the link to the electronic supplementary material.

Supplementary file1 (PDF 47 kb)

Supplementary file2 (PDF 58 kb)

Supplementary file3 (PDF 60 kb)

Supplementary file4 (PDF 89 kb)

Supplementary file5 (XLSX 178 kb)

Supplementary file6 (XLSX 47 kb)

## Declarations

### Availability of Data and Material

All data presented in this review are available within the article and supplementary material.
