# Supplementary material for: The Metabolic Signature of Cardiorespiratory Fitness: A Systematic Review
Source: Sports Med. 2021 Nov 10;52(3):527–46. doi: 10.1007/s40279-021-01590-y (PMC8891196; doi:10.1007/s40279-021-01590-y)
Supplement: Supplementary file 1 — Supplementary file1 (PDF 47 kb) [file 40279_2021_1590_MOESM1_ESM.pdf]

**Item**	**Specification**
Population or participants and conditions of interest	Humans (any age, any sex and any health condition)
Exposure	Metabolites derived from metabolomics or multi-omics studies applying metabolomics approaches
Comparisons or control groups	NA
Outcomes of interest	CRF measured by means of a cardiopulmonary exercise test (spiroergometry)
Study designs	Any study design, only published studies, no editorials, letters, reviews, meta-analyses, case reports or conference abstracts
CRF: cardiorespiratory fitness	

Supplementary Material 1: The Population, Exposure, Comparison, Outcome, Study design process
