# Supplementary material for: The Metabolic Signature of Cardiorespiratory Fitness: A Systematic Review
Source: Sports Med. 2021 Nov 10;52(3):527–46. doi: 10.1007/s40279-021-01590-y (PMC8891196; doi:10.1007/s40279-021-01590-y)
Supplement: Supplementary file 2 — Supplementary file2 (PDF 58 kb) [file 40279_2021_1590_MOESM2_ESM.pdf]

Search strategies

PubMed

(metabolomics[MeSH] OR metabolome[mesh] OR glycomics[mesh] OR systems biology[mesh] OR metabolomic*[tiab] OR metabolom*[tiab] OR metabonom*[tiab] OR metabolic profil*[tiab] OR metabolic analys*[tiab] OR metabolic analyz*[tiab] OR metabolic signature*[tiab] OR metabolic pathway*[tiab] OR metabolic approach*[tiab] OR metabolic adaption*[tiab] OR metabolic score* [tiab] OR metabolic fingerprint* [tiab] OR metabolism fingerprint* [tiab] OR metabolite fingerprint* [tiab] OR lipidom*[tiab] OR glycom*[tiab] OR multiomic*[tiab] OR multi-omic*[tiab] OR omic*[tiab] OR systems biolog*[tiab]) AND ("cardiorespiratory fitness"[MeSH] OR exercise[MeSH:noexp] OR oxygen consumption[mesh:noexp] OR exercise test[mesh:noexp] OR cardiorespiratory fitness[tiab] OR Vo2peak[tiab] OR Vo2 peak[tiab] OR Vo 2peak[tiab] OR Vo 2 peak[tiab] OR peakVo2[tiab] OR peak Vo2[tiab] OR peak V o2[tiab] OR peakVo 2[tiab] OR peak Vo 2[tiab] OR peak V o 2[tiab] OR peakO2[tiab] OR peak O2[tiab] OR peakO 2[tiab] OR peak O 2[tiab] OR O2peak[tiab] OR O2 peak[tiab] OR O 2peak[tiab] OR O 2 peak[tiab] OR Vo2max[tiab] OR Vo2 max[tiab] OR Vo 2max[tiab] OR Vo 2 max[tiab] OR maxVo2[tiab] OR max Vo2[tiab] OR max V o2[tiab] OR maxVo 2[tiab] OR max Vo 2[tiab] OR max V o 2[tiab] OR maxO2[tiab] OR max O2[tiab] OR maxO 2[tiab] OR max O 2[tiab] OR O2max[tiab] OR O2 max[tiab] OR O 2max[tiab] OR O 2 max[tiab] OR maximal oxygen uptake[tiab] OR maximal o2 uptake[tiab] OR peak oxygen uptake[tiab] OR peak o2 uptake[tiab] OR oxygen peak uptake[tiab] OR o2 peak uptake[tiab] OR maximal oxygen consumption[tiab] OR maximal o2 consumption[tiab] OR peak oxygen consumption[tiab] OR peak o2 consumption[tiab] OR oxygen peak consumption[tiab] OR o2 peak consumption[tiab] OR peak consumption of oxygen[tiab] OR peak consumption of o2[tiab] OR maximal consumption of oxygen[tiab] OR maximal consumption of o2[tiab] OR exercis*[tiab] OR spiroergometry[tiab] OR ergospirometry[tiab] OR spiroergography[tiab] OR ergospirography[tiab] OR CRF[tiab] OR exercise test*[tiab] OR CPET[tiab] OR CPX[tiab] OR CPEX[tiab]) NOT (animals[mesh] NOT humans[mesh])

EMBASE

('metabolomics'/exp OR 'metabolome'/exp OR 'metabolic fingerprinting'/de OR 'glycomics'/exp OR 'systems biology'/exp OR metabolomic*:ti,ab OR metabolom*:ti,ab OR metabonom*:ti,ab OR "metabolic profil*":ti,ab OR "metabolic analys*":ti,ab OR "metabolic analyz*":ti,ab OR "metabolic signature*":ti,ab OR "metabolic pathway*":ti,ab OR "metabolic approach*":ti,ab OR "metabolic adaption*":ti,ab OR "metabolic score*":ti,ab OR "metabolic fingerprint*":ti,ab OR "metabolism fingerprint*":ti,ab OR "metabolite fingerprint*":ti,ab OR lipidom*:ti,ab OR glycom*:ti,ab OR multiomic*:ti,ab OR multi-omic*:ti,ab OR omic*:ti,ab OR "systems biolog*":ti,ab) AND ('cardiorespiratory fitness'/exp OR 'exercise'/de OR 'aerobic exercise'/de OR 'exercise physiology'/de OR 'oxygen consumption'/de OR 'exercise test'/de OR 'ergospirometry'/de OR "cardiorespiratory fitness":ti,ab OR Vo2peak:ti,ab OR "Vo2 peak":ti,ab OR "Vo 2peak":ti,ab OR "Vo peak":ti,ab OR " V o 2 peak”:ti,ab OR peakVo2:ti,ab OR "peak Vo2":ti,ab OR "peak V o2":ti,ab OR "peakVo 2":ti,ab OR "peak Vo 2":ti,ab OR "peak V o 2":ti,ab OR V̇o2peak:ti,ab OR "V̇o2 peak":ti,ab OR "V̇o 2peak":ti,ab OR "V̇o peak":ti,ab OR "V̇ o 2 peak":ti,ab OR peakV̇o2:ti,ab OR "peak V̇o2":ti,ab OR "peak V̇ o2":ti,ab OR "peakV̇o 2":ti,ab OR "peak V̇o 2":ti,ab OR "peak V̇ o 2":ti,ab OR Vo2max:ti,ab OR "Vo2 max":ti,ab OR "Vo 2max":ti,ab OR "Vo max":ti,ab OR " V o 2 max":ti,ab OR maxVo2:ti,ab OR "max Vo2":ti,ab OR "max V o2":ti,ab OR "maxVo 2":ti,ab OR "max Vo 2":ti,ab OR "max V o 2":ti,ab OR V̇o2max:ti,ab OR "V̇o2 max":ti,ab OR "V̇o 2max":ti,ab OR "V̇o max":ti,ab OR "V̇ o 2 max":ti,ab OR maxV̇o2:ti,ab OR "max V̇o2":ti,ab OR "max V̇

o2":ti,ab OR "maxV̇o 2":ti,ab OR "max V̇o 2":ti,ab OR "max V̇ o 2":ti,ab OR "maximal oxygen uptake":ti,ab OR "maximal o2 uptake":ti,ab OR "peak oxygen uptake":ti,ab OR "peak o2 uptake":ti,ab OR "oxygen peak uptake":ti,ab OR "o2 peak uptake":ti,ab OR "maximal oxygen consumption":ti,ab OR "maximal o2 consumption":ti,ab OR "peak oxygen consumption":ti,ab OR "peak o2 consumption":ti,ab OR "oxygen peak consumption":ti,ab OR "o2 peak consumption":ti,ab OR "peak consumption of oxygen":ti,ab OR "peak consumption of o2":ti,ab OR "maximal consumption of oxygen":ti,ab OR "maximal consumption of o2":ti,ab OR exercis*:ti,ab OR spiroergometry:ti,ab OR ergospirometry:ti,ab OR spiroergography:ti,ab OR ergospirography:ti,ab OR CRF:ti,ab OR "exercise test*":ti,ab OR CPET:ti,ab OR CPX:ti,ab OR CPEX:ti,ab) NOT (('animal'/de OR 'animal experiment'/exp OR 'nonhuman'/de) NOT ('human'/exp OR 'human experiment'/de)) NOT [conference abstract]/lim

Web of Science
TS=((metabolomic* OR metabolom* OR metabonom* OR "metabolic profil*" OR "metabolic analys*" OR "metabolic analyz*" OR "metabolic signature*" OR "metabolic pathway*" OR "metabolic approach*" OR "metabolic adaption*" OR "metabolic score*" OR "metabolic fingerprint*" OR "metabolism fingerprint*" OR "metabolite fingerprint*" OR lipidom* OR glycom* OR multiomic* OR multi-omic* OR omic* OR "systems biolog*") AND ("cardiorespiratory fitness" OR Vo2peak OR "Vo2 peak" OR "Vo 2peak" OR "Vo2 peak" OR peakVo2 OR "peak Vo2" OR "peak V o2" OR "peakVo 2" OR "peak Vo 2" OR "peak V o 2" OR peakO2 OR "peak O2" OR "peakO 2" OR "peak O 2" OR O2peak OR "O2 peak" OR "O 2peak" OR "O 2 peak" OR Vo2max OR "Vo2 max" OR "Vo 2max" OR "Vo2 max" OR maxVo2 OR "max Vo2" OR "max V o2" OR "maxVo 2" OR "max Vo 2" OR "max V o 2" OR maxO2 OR "max O2" OR "maxO 2" OR "max O 2" OR O2max OR "O2 max" OR "O 2max" OR "O 2 max" OR "maximal oxygen uptake" OR "maximal o2 uptake" OR "peak oxygen uptake" OR "peak o2 uptake" OR "oxygen peak uptake" OR "o2 peak uptake" OR "maximal oxygen consumption" OR "maximal o2 consumption" OR "peak oxygen consumption" OR "peak o2 consumption" OR "oxygen peak consumption" OR "o2 peak consumption" OR "peak consumption of oxygen" OR "peak consumption of o2" OR "maximal consumption of oxygen" OR "maximal consumption of o2" OR exercis* OR spiroergometry OR ergospirometry OR spiroergography OR ergospirography OR CRF OR "exercise test*" OR CPET OR CPX OR CPEX)) NOT DT=(meeting abstract)
