# Supplementary material for: The Metabolic Signature of Cardiorespiratory Fitness: A Systematic Review
Source: Sports Med. 2021 Nov 10;52(3):527–46. doi: 10.1007/s40279-021-01590-y (PMC8891196; doi:10.1007/s40279-021-01590-y)
Supplement: Supplementary file 3 — Supplementary file3 (PDF 60 kb) [file 40279_2021_1590_MOESM3_ESM.pdf]

No	Description
1	Authors and year of publication
2	Country of study
3	Study design
4	Study population
5	Study population demographics (n, age, sex, body mass index, body fat percentage, physical activity levels, cardiorespiratory fitness, medication)
6	Study completion rate
7	Potential health conditions
8	Tissue sample
9	Sample collection and storage
10	Sampling time and nutritional protocol before sampling
11	Metabolite extraction method
12	Metabolomics analytical technique
13	Quality control used to assess data quality
14	Data processing and metabolite annotation
15	Metabolites associated with CRF
16	CPET protocol and exercise exhaustion criteria
CRF: cardiorespiratory fitness, CPET: cardiopulmonary exercise testing	

Supplementary Material 3: Data extracted from every study included in the review
