# Supplementary material for: The Metabolic Signature of Cardiorespiratory Fitness: A Systematic Review
Source: Sports Med. 2021 Nov 10;52(3):527–46. doi: 10.1007/s40279-021-01590-y (PMC8891196; doi:10.1007/s40279-021-01590-y)
Supplement: Supplementary file 4 — Supplementary file4 (PDF 89 kb) [file 40279_2021_1590_MOESM4_ESM.pdf]

Items	Description
1	Were selection criteria clearly described?
3	Was the type of sample fully described?
4	Were the procedures and timing of biological sample collection with respect to clinical factors described with enough detail?
	4.1. Clinical and physiological factors
	4.2. Diagnostic and treatment procedures.
5	Were handling and pre-analytical procedures reported in sufficient detail and similar for the whole sample? And, if differences in procedures were reported, was their effect on the results assessed?
10	Was the execution of the index test described in sufficient detail to permit replication of the test?
11	Was the execution of the reference standard described in sufficient detail to permit its replication?
15	Were uninterpretable/intermediate test results reported?
16	Is it likely that the presence of overfitting was avoided?

Number in the first column refer to QUADOMICS items

QUADOMICS: an adaptation of the Quality Assessment of Diagnostic Accuracy Assessment (QUADAS) for the evaluation of the methodological quality of studies on the diagnostic accuracy of omics-based technologies(Lumbreras et al., 2008)

Supplementary Material 4: QUADOMICS items applicable to the present work

Lumbreras, B., Porta, M., Márquez, S., Pollán, M., Parker, L.A., and Hernández-Aguado, I. (2008). QUADOMICS: An adaptation of the Quality Assessment of Diagnostic Accuracy Assessment (QUADAS) for the evaluation of the methodological quality of studies on the diagnostic accuracy of '-omics'-based technologies. *Clinical Biochemistry* 41(16)**,** 1316-1325. doi: https://doi.org/10.1016/j.clinbiochem.2008.06.018.
